# Estimates of resource transfer via winged adult insects from the hyporheic zone in a gravel‐bed river

**DOI:** 10.1002/ece3.7366

**Published:** 2021-03-11

**Authors:** Mirza A. T. M. Tanvir Rahman, Junjiro N. Negishi, Takumi Akasaka, Futoshi Nakamura

**Affiliations:** ^1^ Laboratory of Watershed Conservation and Management, Graduate School of Environmental Science Hokkaido University Sapporo Japan; ^2^ Department of Environmental Sciences Jahangirnagar University Dhaka Bangladesh; ^3^ Laboratory of Watershed Conservation and Management, Faculty of Environmental Earth Science Hokkaido University Sapporo Japan; ^4^ Laboratory of Conservation Ecology, Department of Agriculture and Animal Science Obihiro University of Agriculture and Veterinary Medicine Obihiro Japan; ^5^ Laboratory of Ecosystem Management, Research Faculty of Agriculture Hokkaido University Sapporo Japan

**Keywords:** dispersal, Plecoptera, resource transfer, riparian zone, subsurface interface

## Abstract

Hyporheic zone (HZ) locates below the riverbed providing habitat for macroinvertebrates from where the winged adult insects (i.e., hyporheic insects, HIs) emerge and bring out aquatic resources to the riparian zone. This study estimated mean daily flux as dry biomass (BM), carbon (C), and nitrogen (N) deriving from the dominant HI species *Alloperla ishikariana* (Plecoptera, Chloroperlidae) for a 4th‐order gravel‐bed river during the early‐summer to summer periods. We hypothesized that HIs were an important contributor in total aquatic resources to the riparian zone. In 2017 and 2018, we set parallelly (May to August) and perpendicularly (June to October) oriented Malaise traps to catch the lateral and longitudinal directional dispersing winged adults of *A. ishikariana*, and other Ephemeroptera, Plecoptera, Trichoptera, and Diptera from the river and estimated the directional fluxes of them. We further split the directional fluxes as moving away or back to the channel (for lateral) and from down‐ to upstream or up‐ to downstream (for longitudinal). *Alloperla ishikariana* was similar to other Plecoptera species and differed clearly from Ephemeroptera and Trichoptera in directional characteristics of resources flux, suggesting that the extent and directions of HZ‐derived resource transfer depend on taxon‐specific flight behaviors of HIs. Contributions of *A. ishikariana* to the riparian zone in total aquatic C and N transfer seasonally varied and were lower in May (5%–6%) and August (2%–4%) and the highest in July (52%–70%). These conservative estimates largely increased (9% in May) after the supplementary inclusion of Diptera (Chironomidae and Tipulidae), part of which were considered HIs. We demonstrated that HZ could seasonally contribute a significant portion of aquatic resources to the riparian zone and highlighted the potential importance of HZ in nutrient balance in the river‐riparian ecosystem.

## Graphical abstract

This work addressed how much the winged adult flying hyporheic insects could supply resources from the river considering lateral and longitudinal dimensions of the river. Results showed that winged adult hyporheic insects could transfer a significant amount of resource during summer from the river, and thus, the hyporheic zone has the high potentiality to contribute in the resource budget of the river‐riparian ecosystem in the lateral direction of the river.

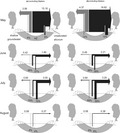

## 1 INTRODUCTION

Macroinvertebrates including insect larvae play vital roles in material cycling and food webs in rivers by contributing to organic matter breakdown and providing, directly and indirectly, food resources for organisms at higher trophic levels (Allan & Castillo, 2007; Macadam & Stockan, 2015). Insect larvae metamorphose into winged adults, leave the water, mate, return to the water, and lay eggs to complete their lifecycle (Williams & Feltmate, 1992) with flight dispersal during the terrestrial stage being a potential bottleneck for population dynamics (Smith et al., 2009). Decades of studies on interactions between rivers and riparian zone also reveal the key role of winged adult aquatic insects in riparian‐river food web because they provide food resources for terrestrial consumers and thereby could contribute to maintaining structure and function of riparian ecosystems as a spatial subsidy (Benjamin et al., 2011; Fukui et al., 2006; Lafage et al., 2019; Nakano & Murakami, 2001; Sabo et al., 2002). For example, birds, bats, spiders, and carabid beetles can depend on aquatic insects at least sometime during the year and this transfer can include transfer of other ecologically important elements (such as mercury) (Walters et al., 2020). Understanding of how and when adult insects disperse, and how much and to where resources are transferred by those vectors can provide critical insights into studies focusing on subsidy and critical life‐cycle stage of insects and ecologically sound management of river‐riparian ecosystems (Muehlbauer et al., 2014; Smith et al., 2009).

River ecosystems are three‐dimensionally structured in space with lateral, longitudinal, and vertical connectivity (Stanford & Ward, 1993). The hyporheic zone, where groundwater mixes with surface water, comprises vertical connectivity and provides a key role in river ecosystems through exchanging matter and heat with surface water, and processing of organic matter and nutrient, and provision of habitat for diverse organisms (Boulton et al., 2010; Robertson & Wood, 2010). This zone can extend vertically and laterally beyond the wetted channel where benthic invertebrates can take refuge during floods, low flow or during dry conditions in the channel (Dole‐Olivier, 2011; Stubbington, 2012; Triska et al., 2018). Besides unique fauna such as stygobites (permanent dwellers of the zone), many larvae of aquatic insects (e.g., Chironomidae, Ephemeroptera, and Plecoptera) are long known to comprise hyporheic invertebrate communities (Astiz & Sabater, 2015; DelVecchia et al., 2016; Pugsley & Hynes, 1986). Their habitat affinity for subsurface domains varies with some spending only a part of their aquatic life in hyporheic zones. In contrast, some species belonging to the order Plecoptera are hyporheic specialists by spending most of their larval life stage in the zone (Dorff & Finn, 2019; Negishi, 2019) (hereafter referred to as “amphibitic insects” or “amphibites”). The increasing number of studies has reported that the relative contribution of hyporheic secondary production to benthic production is substantial within rivers (DelVecchia et al., 2016; Dorff & Finn, 2019; Reynolds & Benke, 2012) whereas winged adults of amphibitic insects are observed in the riparian zones (Negishi, 2019). An emergent but unanswered question is how much contribution the hyporheic zone can provide to the riparian energy budget via dispersal of winged adults of insects including amphibites. Such contributions are predicted to be high in gravel‐bed rivers in particular those characterized with geomorphology that forms relatively deep sediment deposits and high hydraulic conductivity in interstitial space (Stanford & Ward, 1993).

The quantity, timing, direction, and distance of insect‐mediated resource transfer are the function of traits of insects and their abundances (Kovats et al., 1996; Parkyn & Smith, 2011). For instance, large‐sized species with long‐range dispersal ability would provide different characteristics of transferring resources compared to those with relatively small body size and limited dispersal capability. Aquatic insect dispersal patterns have been reported in studies on reproductive behaviors as well as the potential extent of river‐derived subsidy in the riparian zones (Carlson et al., 2016; Muehlbauer et al., 2014). Relatively well‐studied insect taxa include Ephemeroptera (E), Plecoptera (P), and Trichoptera (T) (see, Braun et al., 2014; Lancaster & Downes, 2018; Petersen et al., 2004). The dispersal dimensions of insects relative to emergence point can be crudely decomposed into four: upstream or downstream (i.e., longitudinal dimension) and away from or back to the river (i.e., lateral dimension). Study outcomes are so far mixed but some generalizations can be made. By directly catching near‐ground flying insects, studies have found that adult winged aquatic insects of Plecoptera and Ephemeroptera may travel less and stay close to natal waterbodies whereas Trichoptera may travel much more (Kuusela & Huusko, 1996; Petersen et al., 2004; Winterbourn et al., 2007). Some species exhibit flight over several 100 m to kilometers and/or clear directional flights (Winterbourn et al., 2007; Winterbourn & Crowe, 2001). Direct catches considering vertical dimensions of riparian forest and population genetics studies and dispersal estimates using stable isotopes tend to support longer‐range dispersals especially in a lateral direction (Briers et al., 2004; Chaput‐Bardy et al., 2008; Didham et al., 2012; Macneale, Peckarsky, & Likens, 2004, 2005). Taken together, there might exist species‐ and individual‐specific dispersal patterns related to variations in their strategy for mating and flight modes. Therefore, taxon‐specific resource transfers in multiple dimensional axes would improve our appreciation of mechanisms behind hyporheic‐riparian resource linkages.

This study estimated resource transfer in terms of matter (as dry biomass, BM), energy (as carbon, C), and nutrient (as nitrogen, N) originating from benthic and hyporheic zones to the riparian zone during the earlier‐summer to summer periods (May to August) in a gravel‐bed river. These periods correspond with the timing of the highest abundance of emergent aquatic insects in the study region (Nakano & Murakami, 2001). The study extends on previous studies on aquatic larvae of insects and the identification of amphibitic insect taxa in the study river. Negishi, Hibino, et al. (2019) found one species of Plecoptera belonging to the family Chloroperlidae (*Alloperla ishikariana*) mostly in the hyporheic zone (see also Alam et al., 2020), and this species dominated hyporheic insect biomass comprising >70% (unpublished data, JN Negishi). Species of Chloroperlidae are widely adapted to habitat in the hyporheic zone (e.g., McElravy & Resh, 1991; Ray et al., 2010; Silveri et al., 2009), and thus, we focused on *A. ishikariana* as a model organism to estimate resource transfer associated with insects found in hyporheic zone (i.e., hyporheic insects, HIs). To understand resource transfer in relation to biological traits of contributing HIs, we quantified dispersal movements of winged adults of *A. ishikariana* as well as other insects of EPT taxa in two dimensions (lateral and longitudinal) and quantified directional components in each. Although Diptera (D) can be a significant contributor of resource transfers to riparian zone (Carlson et al., 2016; Jonsson & Stenroth, 2016), preliminary sample observations suggested that this was not the case in our study and thus D was not included as major targeted taxa in this study. To check the validity of this assumption, we also reported estimates only in the lateral dimension including D, by also considering that a part of D comprises HIs (Negishi, Hibino, et al., 2019). We hypothesized that HIs would be an important contributor to total aquatic resources to the riparian zone. We predicted that *A. ishikariana* would provide the resource of a comparable amount to that from other benthic dwellers because our previous study reported abundant *A. ishikariana* in the riparian zone during summer months (Negishi, 2019).

## 2 MATERIALS AND METHODS

### 2.1 Study site

**FIGURE 1:**
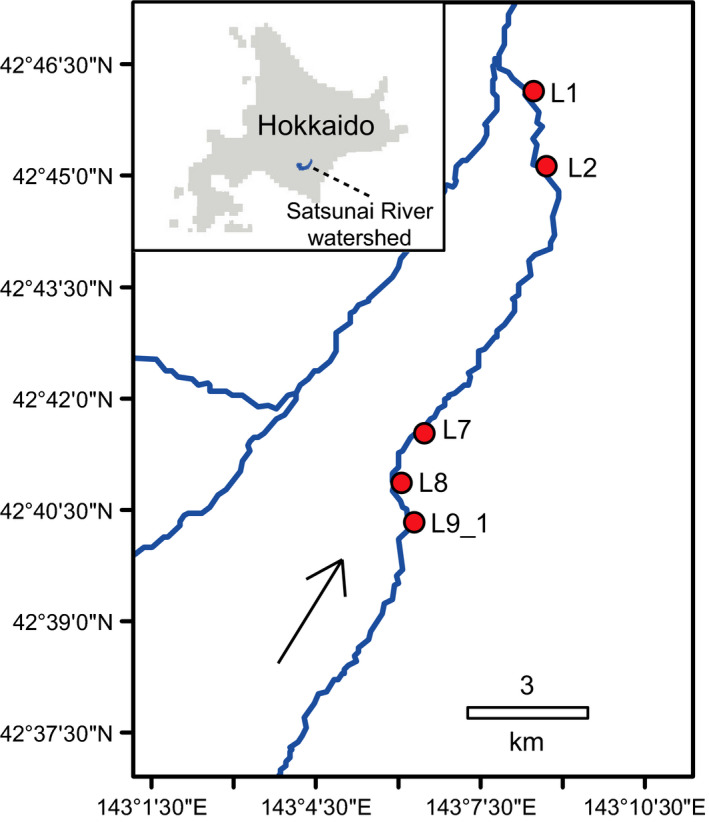
Map of the study area showing the location of the Satsunai River in Hokkaido (a) and the study section (b). Filled circles denote the sampling sites

The study was conducted in the lower catchment of the Satsunai River, which is situated in the south‐eastern part of Hokkaido in Japan having a 725 km^2^ catchment area with a length of 82 km (Figure 1). The regional climate is characterized by low air temperature in winter, which occasionally reaches the lowest of −30°C, and relatively low annual precipitation (the annual total amount usually <1,000 mm). The Satsunai River is a 4th‐order river and the study segment had active low‐flow channels comprised of multiple river channels interspersed with islands and bank‐connected exposed gravel bars. The thickness of the deposited sediment was commonly more than 20 m. The segment had flood protective levees on both sides, with the interlevee width of the channel extending over approximately 350 m. The area between an active channel and the levees consisted of riparian forests dominated by plants such as *Salix* spp. and *Reynoutria japonica*. Spur dikes to control flood and riverbed erosion existed within the segment (Yamaguchi et al., 2013), and there was a multi‐purpose dam to generate hydroelectricity and control floods approximately 25 km upstream from the studied segment (Takahashi & Nakamura, 2011). The hydrograph in typical years was generally characterized by relatively high sustained flow rates for the period between late March and early June because of runoff from snowmelt and occasional floods in late summer and autumn, which still resembles natural seasonal fluctuation of flow in the region. The mean daily flow rate from 2006 to 2016 was approximately 8.6 m^3^ s^−1^ at the Kamisatsunai gauging station of the Ministry of Land, Infrastructure, Transport, and Tourism (MLIT) located within the study segment. More details of the study area were provided in Negishi, Hibino, et al. (2019) and Negishi, Terui, et al. (2019).

### 2.2 Sampling and processing of samples

We used three types of Malaise traps to catch flying insects: Hanging Malaise (HM) trap (length‐200, width‐195, height‐170 cm; Urabe Kagaku Inc., Japan), Single‐headed Malaise (SM) trap (length‐165, width‐180, height‐180 cm; Megaview Science Co., Taiwan), and Double‐headed Malaise (DM) trap (length‐180, width‐180, height‐180 cm; Megaview Science Co., Taiwan) (Appendix S1). Malaise traps have been commonly used to study directional flights of aquatic insects (e.g., Briers et al., 2002; Griffith et al., 1998; Petersen et al., 1999, 2004). A partitioning screen hangs to the central part of the trap guiding insects to the upper end where 75% ethanol‐filled preservation bottle(s) are attached. Single‐headed Malaise and HM traps collect insects from both sides and preserve them together in single 75% ethanol‐filled bottles because the upper corner end of the screen is partially absent. Double‐headed Malaise trap has two bottles each of which is connected to one side of the upper end of a fully closed partitioning screen so that insects from two sides are collected separately.

We set traps in two orientations relative to the river water flow direction. When the traps were set in parallel to the river, we assumed that traps caught flying insects along the lateral dimension that came from the river moving to the riparian forest and (or) from the forest to move back to the river (i.e., lateral movements). When the traps were set perpendicular to the channel, we assumed that traps caught flying insects that moved along the longitudinal dimension from upstream to downstream and (or) from downstream to upstream (i.e., longitudinal movements) (Appendix S1). However, it was possible that parallelly set traps caught insects that traveled longitudinally along the channel over some distances, and thus, the insect abundance estimated in parallelly set traps could partially include those quantified in perpendicularly set traps. It was similarly possible for perpendicular set traps to partially catch those quantified in parallelly set traps. However, the latter case should not be common because the perpendicularly set traps were relatively away from the edge of the forest (>4 m) where parallelly set traps quantified insect abundance.

In order to quantify lateral movements, we set five SM traps in parallel to the river in L1, L2, L7, L8, and L9_1 sites at the edge of the river in the riparian forest from May to August in 2017 and 2018 (Figure 1, Appendix S1). Traps were placed on the ground surface, and thus, the lower end of the partitioning screen was attached to the ground. Samples were collected for a sampling duration of 3–30 days with a sampling frequency of 9–10 times for each trap in 2017 and 4–22 days for 13 times for each trap in 2018 (Appendix S2). SM traps were continuously placed except for some days when the high‐flow events were predicted.

In order to quantify longitudinal movements, we set four HM traps within 200 m of parallelly oriented traps to each of L1, L2, L7, and L8 sites perpendicular to the channel on the water surface of the channel edge from June to October in 2017 and 2018 (Figure 1, Appendix S1). We used HM traps because they could be set above the water surface by being hung using ropes (the bottom of the partitioning screen was 30–50 cm above the water surface) whereas SM and DM traps required a solid platform to be set up stably. Samples were collected for a sampling duration of 7 days with a sampling frequency of 5 times for each trap in 2017 and 3–10 days for 8–9 times for each trap in 2018 (Appendix S2). Hanging Malaise traps were installed when the water level was at the base‐flow condition.

We used side‐wise trapped insects records of DM traps to estimate upstream and downstream portions in longitudinally moving individuals in HM traps. Two DM traps were set perpendicular to the river in L2 and L9_1 sites at the edge of the river in the riparian forest, and one in L8 site on the gravel bar in June 2018 (Figure 1, Appendix S1, Appendix S3). When the insects were caught in the downstream side of the trap, we assumed that they flew from the downstream to the upstream direction and vice versa.

We identified all trapped insects belonging to the three orders (Ephemeroptera—E, Plecoptera—P, and Trichoptera—T). Diptera were not the main targeted taxon and were sorted from randomly selected samples for laterally oriented traps due to cost and labor limitations (Appendix S2). Then from Plecoptera, we further identified *A. ishikariana* and from Diptera, Chironomidae, and Tipulidae. Non‐hyporheic Plecoptera (non‐hyporheic P) referred to Plecoptera without *A. ishikariana*, unless otherwise noted. Among Diptera, we only sorted out Chironomidae and Tipulidae because they were dominating Diptera in the hyporheic zone of the study area according to Negishi, Hibino, et al. (2019).

### 2.3 Estimation of resource transfer

In order to estimate BM, C, and N of E, non‐hyporheic P, T, D, and *A. ishikariana*, we used family‐level mean individual BM (Appendix S4) and community composition of half monthly window of adult aquatic insects in SM trap and HM trap samples (Appendix S5). We calculated the half month window‐wise taxon‐specific mean BM (Appendix S6) by applying relative‐abundance‐based weightings to family‐level mean individual BM in samples collected in 2017, 2018, and 2019 (Appendix S4, Appendix S5). This step for Diptera was performed only for parallelly set traps. The half month window‐wise taxon‐specific mean BM was multiplied by the corresponding window‐wise insect abundances for parallelly and perpendicularly set traps in 2017 and 2018. Percentages of C and N of the dried insects collected between 2015 and 2018 for other studies in the same sites (E: 536 individuals, P: 136, T: 306, D: 48, *A. ishikariana*: 119; JNN, unpublished data) were determined in the isotope ratio mass spectrometer coupled with an elemental analyzer (Model‐Finnigan MAT252; Thermo Fisher Scientific, Florence, United States), and species‐level mean for *A. ishikariana* and order‐level or family‐level means for others were applied to convert BM to C and N for each taxon. Individual‐based C to N ratios were also obtained from the percentages of C and N for a subset of these individuals (E: 76 individuals, P: 37, T: 69, D: 48, *A. ishikariana*: 119).

We quantified the amount of resource transfer in the form of a flow of BM, C, and N in the unit area and time, that is, flux (mg m^−2^ day^−1^). Daily flux was estimated using the following equation: Daily flux = [BM or C or N (mg)] × [trapping area (m^2^) × sampling duration (day)]^−1^. Here, trapping area is the estimated total area of partitioning screen of traps attaching to each preservation bottle. For SM and HM traps, both sides of the screen surface were counted whereas for DM trap one side was counted.

We further decomposed lateral and longitudinal fluxes into two‐directional portions (away and back for lateral, and upstream and downstream for longitudinal). For the longitudinal components, based on the data obtained in DM traps, we estimated mean proportions (%) of the upstream and the downstream moved individuals for each taxon (Appendix S3) and applied it to daily flux of HM traps. For the lateral components, we used previous reports on how much biomass was returning to the channel (Francis et al., 2006; Gray, 1989; Jackson & Fisher, 1986; Stagliano et al., 1998). These estimates overall included 13 out of 15 families found in the Satsunai River (Appendix S4 and Appendix S7). We acquired the returning rate (%) data for each taxa from each of the studies [except for Gray (1989) where we were able to get one mean returning rate for all orders] and pooled together the order‐level data and calculated the mean returning rates for E, P, and T (Appendix S7). In these studies, there was no information on the returning rates of *A. ishikariana*. Therefore, we used the estimated returning rate of P to represent the returning rate of *A. ishikariana*.

Since we used two different traps with different measurements and types of materials and structures (i.e., HM and SM traps), differences in catching efficiency were considered as a potential error source in the flux estimates (Lamarre et al., 2012). A preliminary testing clearly showed that there were differences in catch efficiency (Appendix S8). To correct this and enable comparisons of fluxes in multiple dimensions, we developed two *trap conversion factors* for each taxon (Appendix S8), each of which allowed us to convert the insect abundances of HM traps to be comparable to those from SM traps (factor *a*), and to convert the insect abundances of SM traps to be comparable to those from HM traps (factor *b*). By using these two approaches for adjusting the abundances caught by both traps, we had two values for the estimated fluxes. Therefore, we reported two values as a potential range of the fluxes associated with trap types.

We lastly quantified the amount of transferred resources to the riparian zones deriving from the hyporheic zone relative to overall resources from the river. We reported the contrasts for C and N as mean daily fluxes for May, June, July, and August in moving‐away direction of lateral dimension for hyporheic *A. ishikariana* and all other EPT taxa together collected from SM traps. We reported two sets of results based on two conversion factors. To check how the exclusion of D would affect the estimation, supplementary estimates were recalculated by including D as HIs. To specify D that might have originated from the hyporheic zone (hyporheic D) and benthic zone (non‐hyporheic D), we divided the fluxes of Chironomidae and Tipulidae by considering 50% and 10% of them were from the hyporheic zone according to the information provided in Negishi, Hibino, et al. (2019). Then, we summed the family‐level data to obtain the benthic and hyporheic zones originating Diptera mediated fluxes. We also quantified the fluxes that could be returned to the river by using the mean returning rates based on past studies (Jackson & Fisher, 1986; Gray, 1989; see Appendix S7).

### 2.4 Analyses

We used functions in the “glmmADMB” and “multcomp” packages in R (Version 3.3.2; R Core Team, 2016) to perform the statistical analyses below. The significance level (*α*) was set at *p* = .05.

We ran the generalized linear mixed models (GLMMs) to test the seasonal changes in abundance of insects in relation to taxon identities (E, non‐hyporheic P, T, and *A. ishikariana*) separately for lateral and longitudinal dimensions. We developed a full model with taxon identity and sampling month as main factors and their interaction term. The response variable for all models was insect abundance and biomass (dry mass) in SM or HM traps (a total of four full models). We also included sampling year, sites, and sample collection date as random effects, and the product of sampling duration and trapping area as an offset term to correct for catch efforts. We adopted negative binomial error distribution with a log link function and Gaussian error distribution with identity link function for abundance and biomass, respectively. Biomass was subjected to log_10_ (*x* + 0.01) transformation to improve the normality. Sampling months were assigned according to the median day of each trapping period. To examine whether the seasonal changes differed among the taxon identities, we compared these models with reduced models that excluded the interaction terms using likelihood‐ratio tests. When there were significant differences, we conducted multiple comparisons among taxa separately for each month. In multiple comparisons, *α* was adjusted using Bonferroni correction and divided by the number of months (*p* = .0125 for SM traps and *p* = .01 for HM traps).

To compare dispersal patterns of *A. ishikariana* with other taxa by considering all the directional movements, we further developed GLMMs that included taxon identity and direction as main factors and their interaction term with Gaussian error distribution with the identity link function. The response variables were abundance and biomass for all models, which were adjusted considering trap conversion factors and log_10_ (*x* + 0.01) transformed for June, July, and August when both SM and HM traps data were available. We included sampling year, sites, and sample collection date as random effects. The developed models were compared with the corresponding reduced model that excluded interaction terms by likelihood‐ratio tests. If there was no statistical significance, we interpreted it as no differences between *A. ishikariana* and other taxa in their dispersal patterns relative to directions. When there were significant differences, we conducted multiple comparisons among directions separately for each taxon. In multiple comparisons, *α* was adjusted using Bonferroni correction and divided by the number of directions (*p* = .0125). Since the trap conversion factors for each taxon were used in two approaches for adjusting the catches between SM and HM traps (as described in the previous section), we analyzed twice with the same model structures.

To compare the nutritional quality as potential prey for consumers among E, non‐hyporheic P, T, D, and *A. ishikariana*, we developed another GLMM. The response variable was C to N ratios with log_10_ (*x* + 0.01) transformation, the explanatory variable was taxon identity, random effects were sampling year, site, and sample collection date, and the error distribution was Gaussian with identity link function. The effect of taxon identity was tested by comparing the full model with a null model (without the explanatory variable) using likelihood‐ratio test. When there was a significant difference, we conducted multiple comparisons among all taxa.

## 3 RESULTS

A total of 2,193 and 82 *A. ishikariana*, 42 and 22 E, 2,797 and 276 non‐hyporheic P, and 2,077 and 2,864 T were caught in parallelly oriented SM traps and perpendicularly orientated HM traps, respectively, in 2017 and 2018. From randomly selected samples collected in 2017 and 2018, we counted 1,289 Chironomidae and 9 Tipulidae. HM traps were higher in trapping efficiency when compared at a unit‐area basis (Appendix S8).

**FIGURE 2:**
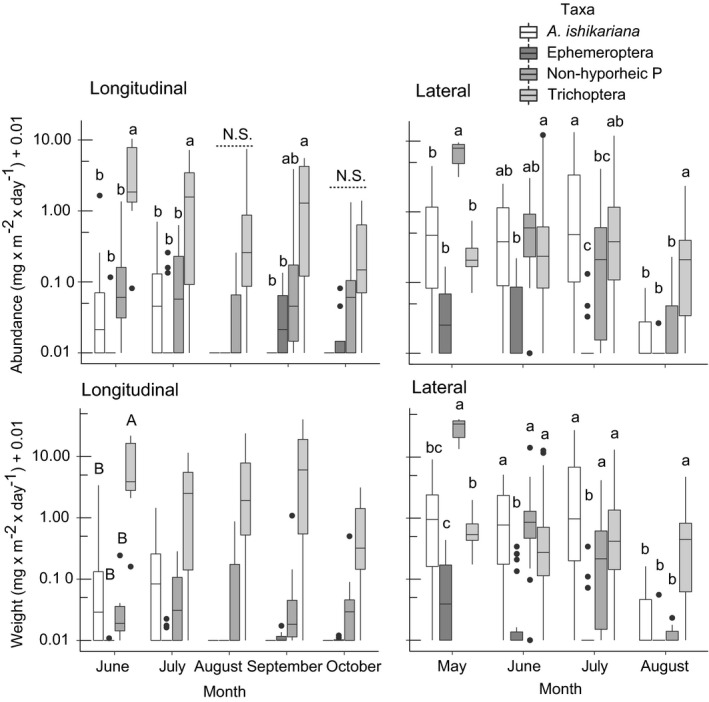
Abundance (upper panels) and biomass (lower panels) of adult aquatic insects in different taxa using traps set in perpendicular (longitudinal) or in parallel (lateral) relative to the river channel. Note that raw data (without using trap conversion factors) are on vertical axes with a logarithmic scale. Those accompanied by the same letter were not statistically different based on the results from multiple‐comparison tests. N.S. denotes that there were no differences among taxa in the corresponding month. Small letters denote the results of comparisons of pairs within each month whereas capital letters denote the results of comparisons of pairs across all months as there was no interaction between taxonomic identity and sampling month (see Table 1). Non‐hyporheic P: Plecoptera without *Alloperla ishikariana*. Boxplot legend: top (bottom) edges of box are 75th (25th) percentiles; center line in the box is median; the upper (lower) whisker extends from the box edge to the largest (smallest) value no further than 1.5 × inter‐quartile ranges of the edge; data beyond the end of the whiskers are outliers and are plotted individually

In both HM and SM traps, the differences in the abundance of each taxon varied across seasons as indicated by the interaction term between taxon identity and month (Table 1). In longitudinal direction (for HM traps), T was among the most abundant in June, July, and September compared with other taxa except for August and October when there were no differences among taxa (Figure 2). In lateral direction (for SM traps), there was a clear temporal shift in numerical dominance of taxa with non‐hyporheic P being the most abundant in May, *A. ishikariana* being among the most abundant in June and July, and T being most abundant in August (Figure 2). Biomass showed similar but less clear differences among taxa or seasons. In the longitudinal direction, only a difference among taxa was detected (Table 1), with T being the highest across seasons (Figure 2). In the lateral direction, non‐hyporheic P had the highest biomass among other taxa in May; in other months, taxa with high abundances also had high biomasses (Figure 2). Ephemeroptera had consistently the lowest biomass in all months reflecting that the total catch was very low.

**TABLE 1:** Summary statistics of the likelihood‐ratio test to examine the effects of interaction between taxonomic identity (Taxa, T) and season (Month, M) on abundance (No. m^−2^ day^−1^) and biomass (mg m^−2^ day^−1^) of flying adults of aquatic insects

Response variable	Direction of flight	Model type	Explanatory variables	*df*	Log‐likelihood	Deviance	*p*‐value
Abundance	Longitudinal	Full	Taxa, Month, T × M	12	−441.63	48.32	**<.001**
		Reduced	Taxa, Month		−465.79		
	Lateral	Full	Taxa, Month, T × M	9	−1,203.1	133.64	**<.001**
		Reduced	Taxa, Month		−1,270.0		
Biomass	Longitudinal	Full	Taxa, Month, T × M	12	−589.26	2.93	.996
		Reduced	Taxa, Month		−590.72		
		2nd reduced	Month	3	−608.25	37.98	**<.001**
		2nd reduced	Taxa	4	−591.94	5.36	.994
	Lateral	Full	Taxa, Month, T × M	9	−749.39	88.90	**<.001**
		Reduced	Taxa, Month		−793.84		

Note

Traps were oriented in parallel or perpendicular to the river channel to estimate lateral and longitudinal dispersing insects, respectively. Comparisons were made between full models and first‐order reduced models without interaction terms (T × M) first. When interaction term was found insignificant, second‐order reduced models (2nd reduced), which included either of two main factors, were separately tested against the first‐order models to examine the effects of main factors. For all models, random effects: sampling year, site, sample collection date; offset term: sampling duration, trapping area; error distribution: negative binomial (for abundance) and Gaussian (for biomass) with log and identity link functions, respectively. Biomass was log_10_ (*x* + 0.01) transformed to improve its normality. *p*‐value is bold when it is significant.

**FIGURE 3:**
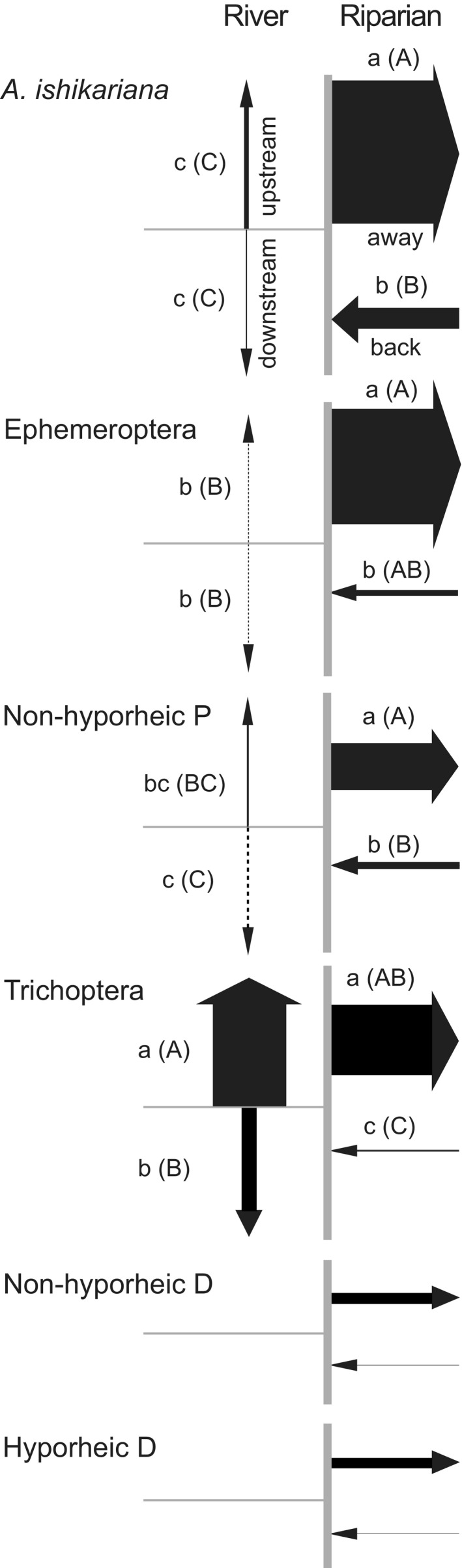
Graphical representation of daily mean fluxes as dry biomass (BM) of Ephemeroptera, non‐hyporheic P (i.e., Plecoptera without *Alloperla ishikariana*), *A. ishikariana* and Trichoptera in longitudinal and lateral directions relative to the river and the riparian zone, and non‐hyporheic Diptera (non‐hyporheic D) and hyporheic Diptera (hyporheic D) in lateral direction using data from June, July, and August. Arrow widths are described as proportionate to relative fluxes within and among taxa; raw values for Ephemeroptera were too small to show relative to other taxa, and thus, arrow widths were exaggerated by multiplying 100% for clarity. Only the cases using conversion factor *a* for longitudinal direction and without conversion for lateral direction were shown; see the text and Table 2B for other results with and without conversion factor. Small letters showing directional statistical differences for each taxon are for the case when hanging Malaise trap catches were adjusted by using trap conversion factor *a* and single‐headed Malaise trap catches were not adjusted; capital letters in the parentheses are for opposite case. *Alloperla ishikariana* demonstrated directional fluxes comparable with non‐hyporheic P. No statistical analysis was done for Diptera

Directional fluxes of the insect in terms of abundance and associated biomass varied among taxa when examined separately in two‐directional portions (Table 2). Longitudinally, T exhibited the highest movements compared with other taxa in both upstream and downstream directions regardless of using conversion factors or not. Laterally, *A. ishikariana* showed the highest values in both away and back directions compared with other taxa when we did not use conversion factor. Diptera from both the benthic and hyporheic zones and T had the second highest abundance away from the river, but the flux of D substantially decreased on a basis of biomass largely due to their small individual biomass (Appendix S4). When using the conversion factor b, the highest value was observed for T away from the river, with *A. ishikariana* being the second highest. These taxon‐specific variations in four directional fluxes were reflected in the presence of significant interaction term between taxon identity and direction (Table 3). *Alloperla ishikariana* exhibited directional fluxes comparable with non‐hyporheic P (Table 3) with disproportionately greater fluxes in lateral portion and the largest amount moving away from the river (Figure 3).

**TABLE 2:** Daily mean (±*SD*) fluxes as abundance and biomass of Ephemeroptera, non‐hyporheic Plecoptera without *Alloperla ishikariana* (non‐hyporheic P), Trichoptera, *A. ishikariana* in longitudinal and lateral directions, and non‐hyporheic Diptera (non‐hyporheic D), hyporheic Diptera (hyporheic D) in lateral direction for June, July, and August

Taxa	Longitudinal		Lateral	
	Upstream	Downstream	Away	Back
	*a*	*a*	*Without conversion*	*Without conversion*
(A)				
Abundance (No. m^−2^ day^−1^)				
Ephemeroptera	<0.00 (<0.00)	<0.00 (<0.00)	0.02 (0.04)	<0.00 (<0.00)
Non‐hyporheic P	0.05 (0.10)	0.01 (0.01)	0.53 (0.73)	0.08 (0.11)
Trichoptera	0.50 (0.65)	0.10 (0.13)	1.04 (2.23)	0.07 (0.15)
Non‐hyporheic D	–	–	1.05 (1.14)	0.05 (0.05)
*A. ishikariana*	0.04 (0.13)	0.01 (0.03)	1.20 (2.28)	0.18 (0.33)
Hyporheic D	–	–	1.03 (1.14)	0.05 (0.05)
Biomass (mg m^−2^ day^−1^)				
Ephemeroptera	<0.00 (<0.00)	<0.00 (<0.00)	0.02 (0.07)	<0.00 (<0.00)
Non‐hyporheic P	0.03 (0.07)	0.005 (0.01)	0.81 (1.61)	0.12 (0.23)
Trichoptera	1.27 (1.57)	0.26 (0.32)	1.22 (2.42)	0.03 (0.06)
Non‐hyporheic D	–	–	0.19 (0.20)	0.01 (0.01)
*A. ishikariana*	0.08 (0.27)	0.02 (0.05)	2.48 (4.73)	0.36 (0.69)
Hyporheic D	–	–	0.18 (0.19)	0.01 (0.01)

Taxa	Longitudinal		Lateral	
	Upstream	Downstream	Away	Back
	*Without conversion*	*Without conversion*	*b*	*b*
(B)				
Abundance (No. m^−2^ day^−1^)				
Ephemeroptera	0.01 (0.02)	0.01 (0.04)	1.60 (3.99)	0.04 (0.10)
Non‐hyporheic P	0.11 (0.22)	0.01 (0.03)	1.14 (1.59)	0.17 (0.23)
Trichoptera	1.92 (2.51)	0.39 (0.51)	3.99 (8.56)	0.26 (0.57)
*A. ishikariana*	0.08 (0.24)	0.02 (0.05)	2.22 (4.23)	0.32 (0.62)
Biomass (mg m^−2^ day^−1^)				
Ephemeroptera	<0.00 (<0.00)	<0.00 (<0.00)	2.33 (7.15)	0.06 (0.17)
Non‐hyporheic P	0.07 (0.15)	0.01 (0.02)	1.77 (3.49)	0.26 (0.51)
Trichoptera	4.88 (6.06)	0.99 (1.23)	4.70 (9.30)	0.11 (0.22)
*A. ishikariana*	0.16 (0.50)	0.03 (0.10)	4.60 (8.75)	0.67 (1.28)

Note

In (A), results in *a* were obtained when hanging Malaise trap catches were adjusted to compare with single‐headed Malaise trap catches using conversion factor *a* and single‐headed Malaise trap catches did not adjusted; and in (B), results in *b* were obtained when single‐headed Malaise trap catches were adjusted to compare with hanging Malaise trap catches using conversion factor *b* and hanging Malaise trap catches did not adjusted. *Without conversion* is mentioned when no conversion factor was used in the estimation.

**TABLE 3:** Summary of likelihood‐ratio tests to examine the interaction between taxonomic identity (Taxa, T) and flight directions (i.e., upstream and downstream directions longitudinally along the channel, and moving away from and toward to the channel laterally on abundance and biomass) (Direction, D)

Response variable	Model type	Explanatory variables	*df*	Log‐likelihood		Deviance		*p*‐value	
				*a*	*b*	*a*	*b*	*a*	*b*
Abundance	Full	Taxa, Direction, T × D	9	−865.84	−1,192.6	117.9	110.16	**<.001**	**<.001**
	Reduced	Taxa, Direction		−924.79	−1,247.7				
Biomass	Full	Taxa, Direction, T × D	9	−973.32	−1,258.3	293.92	229.04	**<.001**	**<.001**
	Reduced	Taxa, Direction		−1,120.28	−1,547.1				

Note

Comparisons were conducted between *Alloperla ishikariana* (as hyporheic insects) and other three taxa (i.e., Ephemeroptera, Non‐hyporheic P, and Trichoptera). Non‐hyporheic P denotes plecopteran insects without *A. ishikariana*. All data were for June, July, and August in 2017 and 2018. For all models, random effects: sampling year, site and sample collection date; error distribution: Gaussian with identity link functions. Abundance (No. m^−2^ day^−1^) and biomass (mg m^−2^ day^−1^) were log_10_ (*x* + 0.01) transformed to improve its normality. Results in *a* and *b* were obtained under conditions when hanging Malaise trap catches were adjusted to compare with single‐headed Malaise trap catches using conversion factor *a* and single‐headed Malaise trap catches did not adjusted; and when single‐headed Malaise trap catches were adjusted to compare with hanging Malaise trap catches using conversion factor *b* and hanging Malaise trap catches did not adjusted, respectively. *df* was same for both analyses. *p*‐value is bold when it is significant.

**FIGURE 4:**
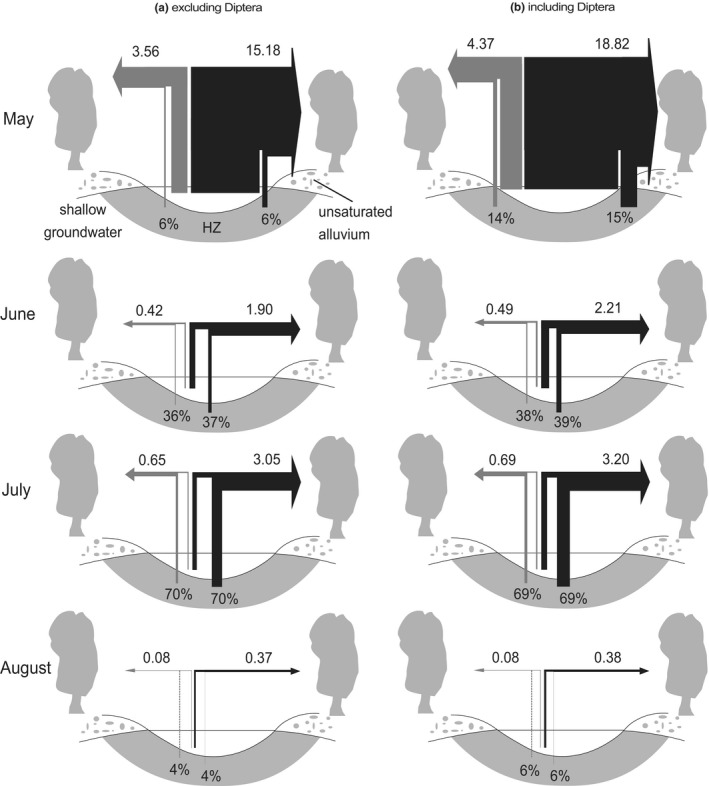
Mean daily fluxes (mg m^−2^ day^−1^) of N (depicted as a gray arrow) and C (depicted as black arrows) transferred from the river to the riparian zone showing relative contribution from the benthic zone and hyporheic zone (HZ) in May, June, July, and August. Results in the left panel (a) excluded Diptera and the right panel (b) included Diptera in calculation. Arrow widths are described as proportionate to relative fluxes within and among taxa. Contributions of the hyporheic zone in resource transfer to the riparian zone were provided in percentages. Only the case using without any conversion factor was shown; see the text for other results on factor *b*

**FIGURE 5:**
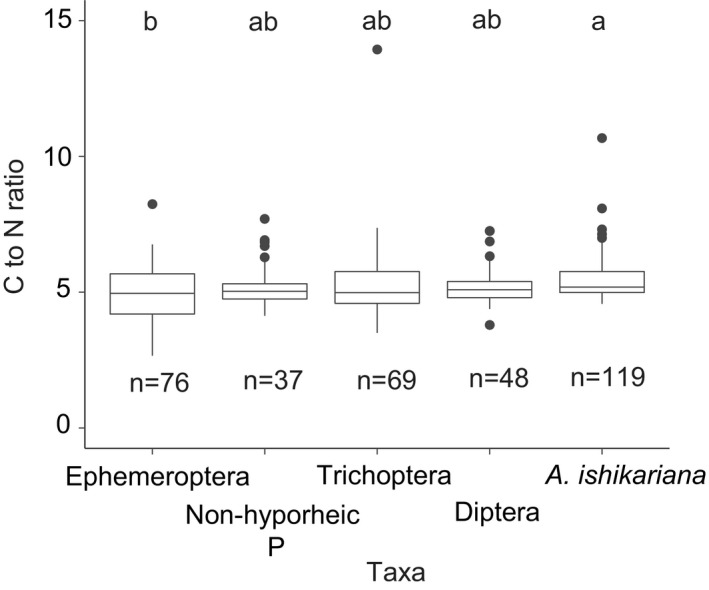
Taxa‐wise C to N ratios. Non‐hyporheic P: Plecoptera without *Alloperla ishikariana*. *n* represents the number of samples. Small letters showing statistical differences among the taxa. For the boxplot legend description, please see Figure 2 caption

Mean daily fluxes of C and N transfer from the river to the riparian zone excluding D was highest in May (C: 15.18–38.72; N: 3.56–8.98 mg m^−2^ day^–1^) and lowest in August (C: 0.37–1.52; N: 0.08–0.31 mg m^−2^ day^−1^) (Figure 4a). Contributions of the hyporheic zone to total C and N transfer seasonally varied with a progressive increase from May (5%–6% for C and 4%–6% for N), June (19%–37% for C and 19%–36% for N) until July (52%–70% for both C and N) and a decrease in August (2%–4% for both C and N). Lower ranges of mean daily flux estimates and upper ranges of contributions of the hyporheic zone in each month were obtained when not using conversion factor and vice versa when using the conversion factor *b*. Supplementary analyses including Diptera increased fluxes in C and N to riparian zone by 4%–24% and 3%–23%, respectively, and largely increased the contributions of the hyporheic zone to total C and N transfer (Figure 4b). The greatest increase was observed in May by 9% for both C and N from the hyporheic zone. Taxa differed in their C to N ratios (*p* < .001; taxon identity effects in likelihood‐ratio test), and *A. ishikariana* was among the highest (Figure 5).

## 4 DISCUSSION

Winged adult aquatic insects have been well‐studied for the understanding of the terrestrial life‐history stage, which is critically important for their population dynamics, as well as potential prey for terrestrial consumers in the recipient riparian zone (Muehlbauer et al., 2014; Smith et al., 2009). Our study extends previous findings that demonstrated the high contribution of hyporheic zone to invertebrate secondary production in rivers (Collier et al., 2004; Dorff & Finn, 2019; Reynolds & Benke, 2012; Smock et al., 1992; Wright‐Stow et al., 2006). Instead of measurements of in‐stream secondary production, we measured resource transfer that could reach a recipient system and provided results supportive of our predictions. Our estimate revealed that the hyporheic zone became a seasonally dominant contributor to total resource transfer to the riparian zone via the emergence of hyporheic insects. In summer months, hyporheic Chloroperlidae plecopteran insect constitution reached 52%–70% of resource transfer to the riparian zone, a quantity that is potentially important in nutrient balance in the river‐riparian ecosystem. Our study provided one of the first quantitative estimates of insect‐mediated resource linkage connecting vertical and lateral dimensions of river ecosystems.

A relatively high contribution of hyporheic resources to the riparian zone was caused partially by the trait of flight dispersal of the targeted taxa, which was similar to non‐hyporheic plecopteran insects. Regardless of their aquatic habitat, that is, benthic or hyporheic, similar behavioral characteristics in flight dispersal may be common among plecopteran species. Plecopteran insects utilize the riparian forest by this group during terrestrial stages although how far away from rivers they fly could vary among species (Didham et al., 2012; Kuusela & Huusko, 1996; Winterbourn et al., 2007). It is reasonable to expect high variations among different taxa in relative usages of spatial dimensions during flight dispersals of adult aquatic insects (Didham et al., 2012; Petersen et al., 2004; Winterbourn et al., 2007). Thus, the estimates of resources from hyporheic zone to riparian zone likely vary depending on the identity of focal taxa. If hyporheic insects consist of species that exhibit more longitudinally oriented directional flight relative to that in the lateral direction, a less amount of the secondary production deriving from the hyporheic zone might be provided to the riparian zone. Other taxa such as trichopteran species (e.g., *Olinga feredayi*), ephemeropteran species (e.g., *Acanthophlebi cruentata*), and nonbiting midge larvae of chironomid insects can also inhabit the hyporheic zone (Collier et al., 2004; Descloux et al., 2013; Reynolds & Benke, 2012; Wright‐Stow et al., 2006). If flight traits are unique to each taxon irrespective of their habitat affinity with hyporheic zone, these hyporheic trichopteran and ephemeropteran species may disperse via flight in a way very different from that shared by plecopteran species. Specifically, these taxa may disproportionately choose to disperse along river channels rather than into riparian forests (Didham et al., 2012), resulting in fewer resources if they dominate the hyporheic insect community. At least trichopteran insects exhibited such a tendency also in our study. Potential differences in hyporheic to riparian resource linkages associated with taxa‐specific variation suggest that the contribution of hyporheic resources in the recipient system is not necessarily predictable in a linear manner from the secondary production within the river. Proper assessment of the role of hyporheic zone in material cycling involving riparian zone via winged adult insects necessitates the identification of species inhabiting the hyporheic zone and appreciation of their directional flight behavior.

Seasonal variation in aquatic insect‐mediated resource transfer is common in the lotic ecosystem (Nakano & Murakami, 2001; Paetzold & Tockner, 2005; Wesner, 2010). Our seasonal flux estimate agrees with these reports, and the highest estimate in May was caused largely by the emergence of benthic trichopteran and plecopteran insects. It was also apparent that the seasonally variable contribution of hyporheic insects depended on the emergence timing of hyporheic insects. *Alloperla ishikariana* emerged most abundantly in the mid‐summer when the emergence of other taxa was relatively low, leading to a remarkably high contribution. As introduced in the previous paragraph, different taxa may dominate hyporheic insects in other areas. Therefore, if hyporheic insects are characterized by the emergence timing that synchronizes with the emergence timing of numerically and biomass‐wise dominant benthic taxa, for example, the contribution of hyporheic zone might be reduced.

The exclusion of Diptera from main targeted taxa did not overestimate the contribution of HIs to riparian zone. Diptera can be found in the hyporheic zone and in benthic habitat (Carlson et al., 2016; Reynolds & Benke, 2012; Smock et al., 1992) and thus they potentially affect both total flux estimates from rivers as well as the relative proportion of hyporheic invertebrates and consequently, their inclusion or exclusion in estimates could affect estimated flux from the hyporheic zone. For example, along a forest‐to‐agriculture gradient in ten streams the mean biomass of adult Chironomidae was 45 mg m^−2^ day^−1^ during the peak emergence (Jonsson & Stenroth, 2016). In our study site, the effects of Diptera were not strong. The first reason might be the time of mass emergence of adult Diptera which could be different from summer. Several workers reported that the peak emergence of Chironomidae occurred during spring and autumn seasons in low‐temperature streams (Bouchard & Ferrington, 2009; Garcia & Suarez, 2007; Soszyńska‐Maj et al., 2015). This tendency was also found in our study because the inclusion of Diptera changed the estimates mostly in May when the temperature was the lowest during the study period. The second reason was related to the small body size of most captured adult Diptera. Adult Chironomidae which was the dominant Diptera in the hyporheic zone in our study had the lowest mean dry biomass among all insect families.

There were several limitations in our approach for accurately estimating resource transfer from the river to the riparian zone. Firstly, an error could have been involved because our estimate of hyporheic contribution assumed that only one amphibitic Chloroperlidae species dominated the hyporheic zone. This assignment of species was based on the assessment of a benthic and hyporheic invertebrate community in hyporheic traps deployed in the river during the summer period (June to July) (Negishi, Hibino, et al., 2019). It might be possible that some species were not properly assigned because of their life‐cycle characteristics in the hyporheic zone during this period. For example, numerous studies have reported Leuctridae plecopteran species to be hyporheic dependent species (DelVecchia et al., 2016; Dorff & Finn, 2019; Stanford & Gaufin, 1974). We observed the abundant emergence of this group in May (see Appendix S5). If eggs or newly hatched individuals of this taxa were too small to be detected in hyporheic traps during summer months, our current estimate that assigned them being of non‐hyporheic origin was less erroneous. However, this type of habitat affinity assignment errors at least did not distort our arguments about the conservative importance of hyporheic zone. Secondly, we measured only flying insects near the ground. Didham et al. (2012) found that EPT differed in the vertical distribution in the riparian forest with ET more dependent on forest canopy compared to the ground surface. Thus, our estimates should be viewed as those relevant to the near‐ground zone flyer in the riparian forest. Thirdly, our study did not cover all the seasons that limited us from estimating the annual flux.

The functional importance of hyporheic‐originated resource transfer to the riparian zone in recipient consumers as spatial resource subsidy remains unknown. At least, we found that per‐capita nutritional quality of *A. ishikariana* was not better than other benthic dwelling taxa as shown in relatively high C to N ratios although *A. ishikariana* could supply more food for its greater abundance during mid‐summer for the riparian consumers. Previous studies have shown that riparian consumers in riparian zone are dependent on resources provided from the river (Benjamin et al., 2011; Nakano & Murakami, 2001; Paetzold & Tockner, 2005; Terui et al., 2018), with some studies suggesting that the effect size of resource subsidy depends on seasonal context as well as conditions across the boundary (Lafage et al., 2019; Marczak et al., 2007). Our estimates were conducted in the periods extending over mid‐summer when terrestrial productivity also tends to maximize, pointing to the possibility that the dependence of recipient consumers on river resources may not be high. Nevertheless, consumers such as carabid beetles and spiders on exposed gravel bars are abundant in summer and are highly dependent on aquatic resources (Terui et al., 2018). Furthermore, plecopteran species may provide a most preferable food resource for ground‐dwelling consumers because they typically emerge from water by crawling up from the river edge to ground surface with a high chance of an encounter with predatory consumers (Fenoglio et al., 2008; Hynes, 1976). Therefore, although our current estimates focused on the summer period, plecopteran hyporheic insects may provide an important food resource for some riparian consumers. This predator–prey interaction might have reduced *A. ishikariana* individuals reaching to the traps used in this study, and thus needs to be considered also as a potential source of error in our resource underestimates. Despite that numerous previous studies have quantitatively demonstrated trophic interactions between the river and riparian ecosystems, estimates of how resources from rivers can be incorporated into terrestrial food web are disproportionately focused on relatively small forested streams (Lafage et al., 2019). Further food‐web studies on the functional importance of resources deriving from the hyporheic zone would help to synthesize existing views on river‐riparian interactions in relatively large gravel‐bed rivers.

In conclusion, we demonstrated that hyporheic insects are an important contributor to total aquatic resources to the riparian zone at least in summer. The high contribution of the hyporheic zone to the riparian zone was associated with species identity and traits in flight dispersal. Our results underscore the linkages between hyporheic and riparian zone via material transfer by winged adult insects, calling for future works on quantitative assessment of them and functional effects on the recipient food web. Our results also support the notion that focus only on invertebrates in surficial sediments can underestimate secondary production of the stream insect population and overlook the potential functional importance of hyporheic zone in the river‐riparian food web and energy budget. Quantitative estimates of resource exchange in neighboring ecosystems provide fundamental knowledge concerning food‐web interaction at a landscape scale, which is probably the most relevant scale for improved ecologically sound land management practices. Worldwide, despite the suggested functional importance, the hyporheic zone has been degraded and received less attention in river quality assessment as well as restoration efforts compared to other spatial dimensions of rivers (Boulton et al., 2010; Hester & Gooseff, 2010; Kasahara & Hill, 2006). Future studies involving assessment of a hyporheic community in other seasons in multiple river systems would reveal the more complete year‐around significance of the hyporheic zone as a source of resource transferring from the river to the riparian zone and add to the reason for the conservation of hyporheic zone.

## CONFLICT OF INTEREST

Authors declare that there is no conflict of interest to disclose.

## AUTHOR CONTRIBUTIONS

**Mirza A. T. M. Tanvir Rahman:** Conceptualization (equal); data curation (equal); formal analysis (equal); writing–original draft preparation (equal). **Junjiro N. Negishi:** Conceptualization (equal); data curation (equal); formal analysis (equal); funding acquisition (equal); project administration (lead); supervision (lead); writing–original draft preparation (equal). **Takumi Akasaka:** Data curation (equal); writing–review and editing (equal). **Futoshi Nakamura:** Funding acquisition (equal); writing–review and editing (equal).

## ETHICAL APPROVAL

No ethical violation was occurred in this research.

## Supporting information

Appendix S1

Appendix S2

Appendix S3

Appendix S4

Appendix S5

Appendix S6

Appendix S7

Appendix S8

## Data Availability

Data underlying this article are available on the Dryad Digital Repository (https://doi.org/10.5061/dryad.wstqjq2kg).
