# Supplementary material for: Estimates of resource transfer via winged adult insects from the hyporheic zone in a gravel‐bed river
Source: Ecol Evol. 2021 Mar 11;11(9):4656–69. doi: 10.1002/ece3.7366 (PMC8093731; doi:10.1002/ece3.7366)
Supplement: Supplementary file 1 — Appendix S1 [file ECE3-11-4656-s006.docx]

**Supplementary material S1.** Photos of Single-headed Malaise, Hanging Malaise and Double-headed Malaise traps

Photos of installed Single-headed Malaise (SM) trap and Hanging Malaise (HM) trap in the riparian forest (a: SM trap; b: HM trap) and on the gravel bar and the river (c: SM trap; d: HM trap), and Double-headed Malaise (DM) trap on the gravel bar (e). Traps in (a) and (b) were placed in parallel with the river whereas those in (c), (d), and (e) were perpendicular to the channel.
