# Supplementary material for: Estimates of resource transfer via winged adult insects from the hyporheic zone in a gravel‐bed river
Source: Ecol Evol. 2021 Mar 11;11(9):4656–69. doi: 10.1002/ece3.7366 (PMC8093731; doi:10.1002/ece3.7366)
Supplement: Supplementary file 2 — Appendix S2 [file ECE3-11-4656-s007.docx]

**Supplementary material S2.** Summary of winged adults sampling dates and duration

Summary of sampling dates and duration using Single-headed Malaise (SM) traps and Hanging Malaise (HM) traps. Dates (month/day) are shown for median dates in the respective sampling with numbers in brackets being duration in days. Asterisks indicate the sampling events that were randomly selected to count Diptera abundance.

| Trap orientation | Year | Month | Dates |
| --- | --- | --- | --- |
| Longitudinal (HM) | 2017 | June | 6/31 (7) |
| Longitudinal (HM) | 2017 | July | 7/24 (7) |
| Longitudinal (HM) | 2017 | August | 8/24 (7) |
| Longitudinal (HM) | 2017 | September | 9/13 or 9/14 (7) |
| Longitudinal (HM) | 2017 | October | 10/28 (7) |
| Longitudinal (HM) | 2018 | June | 6/23 (7) |
| Longitudinal (HM) | 2018 | July | 7/23 (7); 7/28 (4) |
| Longitudinal (HM) | 2018 | August | 8/5 (10); 8/23 (3); 8/29 (4) |
| Longitudinal (HM) | 2018 | September | 9/2 (4); 9/21 (7) |
| Longitudinal (HM) | 2018 | October | 10/18 (7) |
| Lateral (SM) | 2017 | May | 5/20 (6); 5/26 (7)* |
| Lateral (SM) | 2017 | June | 6/6 (13)*; 6/14 (8); 6/21 (3); 6/25 (4)* |
| Lateral (SM) | 2017 | July | 7/2 (10); 7/8 (3)*; 7/12(4); 7/22 (16)* |
| Lateral (SM) | 2017 | August | 8/15 (30)* |
| Lateral (SM) | 2018 | May | 5/14 (11); 5/27 (17) |
| Lateral (SM) | 2018 | June | 6/9 (5)*; 6/15 (7); 6/21 (4)*; 6/25 (4); 6/30 (5) |
| Lateral (SM) | 2018 | July | 7/4 (4)*; 7/9 (4); 7/13 (5); 7/17 (3); 7/21 (5); 7/24 (3)*; 7/28 (4) |
| Lateral (SM) | 2018 | August | 8/4 (10)*; 8/20 (22)* |
