# Supplementary material for: Estimates of resource transfer via winged adult insects from the hyporheic zone in a gravel‐bed river
Source: Ecol Evol. 2021 Mar 11;11(9):4656–69. doi: 10.1002/ece3.7366 (PMC8093731; doi:10.1002/ece3.7366)
Supplement: Supplementary file 3 — Appendix S3 [file ECE3-11-4656-s002.docx]

**Supplementary material S3.** Calculation of upstream and downstream moved winged adult insects

Calculation of upstream and downstream moved insects (as %) of Ephemeroptera (E), Plecoptera (without *Alloperla ishikariana*) (P), Trichoptera (T), *A. ishikariana* using Double-headed Malaise (DM) traps. DM traps were installed at the river edge in the riparian forest perpendicularly relative to the river in sites L2 and L9_1 and on the bank connected gravel-bar in L8 site on June 2019. All catches were normalized with sampling duration for each taxon, the mean abundances of sidewise catches were determined, and finally the percentages of upstream and downstream moved abundances for each taxon were calculated.

| Starting date | Collection date | Sampling duration (day) | Site | Trap location | Direction of insect movement | Abundance | | | | Abundance/day | | | |
| --- | --- | --- | --- | --- | --- | --- | --- | --- | --- | --- | --- | --- | --- |
|  |  |  |  |  |  | E | P | T | *A. ishikariana* | E/day | P/day | T/day | *A. ishikariana*/day |
| 10-Jun-19 | 13-Jun-19 | 3 | L9_1 | forest-river edge | Downstream | 0 | 9 | 18 | 8 | 0.0 | 3.0 | 6.0 | 2.7 |
| 10-Jun-19 | 13-Jun-19 | 3 | L9_1 | forest-river edge | Upstream | 2 | 50 | 68 | 20 | 0.7 | 16.7 | 22.7 | 6.7 |
| 10-Jun-19 | 13-Jun-19 | 3 | L8 | gravel-river edge | Downstream | 4 | 0 | 40 | 4 | 1.3 | 0.0 | 13.3 | 1.3 |
| 10-Jun-19 | 13-Jun-19 | 3 | L8 | gravel-river edge | Upstream | 0 | 0 | 26 | 1 | 0.0 | 0.0 | 8.7 | 0.3 |
| 10-Jun-19 | 13-Jun-19 | 3 | L2 | forest-river edge | Downstream | 0 | 1 | 12 | 2 | 0.0 | 0.3 | 4.0 | 0.7 |
| 10-Jun-19 | 13-Jun-19 | 3 | L2 | forest-river edge | Upstream | 1 | 0 | 42 | 1 | 0.3 | 0.0 | 14.0 | 0.3 |
| 13-Jun-19 | 14-Jun-19 | 1 | L8 | gravel-river edge | Downstream | 2 | 0 | 14 | 1 | 2.0 | 0.0 | 14.0 | 1.0 |
| 13-Jun-19 | 14-Jun-19 | 1 | L8 | gravel-river edge | Upstream | 0 | 0 | 13 | 0 | 0.0 | 0.0 | 13.0 | 0.0 |
| 13-Jun-19 | 20-Jun-19 | 7 | L9_1 | forest-river edge | Downstream | 0 | 4 | 98 | 1 | 0.0 | 0.6 | 14.0 | 0.1 |
| 13-Jun-19 | 20-Jun-19 | 7 | L9_1 | forest-river edge | Upstream | 0 | 61 | 1198 | 34 | 0.0 | 8.7 | 171.1 | 4.9 |
| 13-Jun-19 | 20-Jun-19 | 7 | L2 | forest-river edge | Downstream | 0 | 0 | 35 | 1 | 0.0 | 0.0 | 5.0 | 0.1 |
| 13-Jun-19 | 20-Jun-19 | 7 | L2 | forest-river edge | Upstream | 5 | 8 | 89 | 5 | 0.7 | 1.1 | 12.7 | 0.7 |
| 20-Jun-19 | 22-Jun-19 | 2 | L9_1 | forest-river edge | Downstream | 0 | 1 | 0 | 1 | 0.0 | 0.5 | 0.0 | 0.5 |
| 20-Jun-19 | 22-Jun-19 | 2 | L9_1 | forest-river edge | Upstream | 0 | 2 | 36 | 5 | 0.0 | 1.0 | 18.0 | 2.5 |
| 20-Jun-19 | 22-Jun-19 | 2 | L8 | gravel-river edge | Downstream | 0 | 0 | 0 | 0 | 0.0 | 0.0 | 0.0 | 0.0 |
| 20-Jun-19 | 22-Jun-19 | 2 | L8 | gravel-river edge | Upstream | 0 | 0 | 16 | 0 | 0.0 | 0.0 | 8.0 | 0.0 |
| 22-Jun-19 | 26-Jun-19 | 4 | L9_1 | forest-river edge | Downstream | 0 | 0 | 8 | 1 | 0.0 | 0.0 | 2.0 | 0.3 |
| 22-Jun-19 | 26-Jun-19 | 4 | L9_1 | forest-river edge | Upstream | 0 | 17 | 54 | 6 | 0.0 | 4.3 | 13.5 | 1.5 |
| 20-Jun-19 | 24-Jun-19 | 4 | L2 | forest-river edge | Downstream | 1 | 0 | 0 | 0 | 0.3 | 0.0 | 0.0 | 0.0 |
| 20-Jun-19 | 24-Jun-19 | 4 | L2 | forest-river edge | Upstream | 0 | 1 | 19 | 3 | 0.0 | 0.3 | 4.8 | 0.8 |
|  |  |  |  |  | Upstream moved mean abundance | | | | | 0.2 | 3.2 | 28.6 | 1.8 |
|  |  |  |  |  | Downstream moved mean abundance | | | | | 0.4 | 0.4 | 5.8 | 0.7 |
|  |  |  |  |  | Total upstream and downstream moved mean abundance | | | | | 0.5 | 3.6 | 34.5 | 2.4 |
|  |  |  |  |  | % of upstream moved | | | | | 32.4 | 87.9 | 83.1 | 72.5 |
|  |  |  |  |  | % of downstream moved | | | | | 67.6 | 12.1 | 16.9 | 27.5 |
