# Supplementary material for: Estimates of resource transfer via winged adult insects from the hyporheic zone in a gravel‐bed river
Source: Ecol Evol. 2021 Mar 11;11(9):4656–69. doi: 10.1002/ece3.7366 (PMC8093731; doi:10.1002/ece3.7366)
Supplement: Supplementary file 4 — Appendix S4 [file ECE3-11-4656-s001.docx]

**Supplementary material S4.** Estimation of taxon-specific dry biomass for winged adults

Taxon-specific dry mass (mg) estimated for individuals of Ephemeroptera, Plecoptera, Trichoptera and Diptera taxa in the Satsunai River, Hokkaido, Japan. Mean, standard deviation (SD) and the number of measured individuals (N) are shown. Samples were collected between March and November in 2015 and 2016. Samples were sacrificed in 75% ethanol and then dried at 60 ˚C for 24 h before measurements. Chloroperlidae without species name refers to taxa other than *Alloperla ishikariana*. **†** indicates the families which were collected in 2018. * indicates the families which were present in the past studies and thus were incorporated in the calculation of the mean returning rate of aquatic insects to the channel (see S7).

| Order | Family | Species | Mean | SD | N |
| --- | --- | --- | --- | --- | --- |
| Ephemeroptera | Baetidae* |  | 1.40 | 1.80 | 39 |
| Ephemeroptera | Caenidae* |  | 0.60 | 0.08 | 2 |
| Ephemeroptera | Ephemerellidae* |  | 1.71 | 1.04 | 18 |
| Ephemeroptera | Ephemeridae |  | 9.48 | 6.75 | 8 |
| Ephemeroptera | Heptageniidae* |  | 2.55 | 1.21 | 12 |
| Ephemeroptera | Leptophlebiidae* |  | 0.59 | 0.28 | 7 |
| Plecoptera | Capniidae* |  | 0.87 | 0.48 | 20 |
| Plecoptera | Chloroperlidae* |  | 1.42 | 0.17 | 3 |
| Plecoptera | Chloroperlidae | *Alloperla ishikariana* | 2.07 | 0.84 | 13 |
| Plecoptera | Leutridae* |  | 0.78 | 0.87 | 9 |
| Plecoptera | Nemouridae |  | 1.14 | 0.57 | 32 |
| Plecoptera | Perlodidae* |  | 17.58 | 14.31 | 13 |
| Trichoptera | Apataniidae |  | 2.14 | 0.94 | 7 |
| Trichoptera | Glossosomatidae |  | 1.59 | 0.99 | 13 |
| Trichoptera | Goeridae |  | 4.25 | 0.43 | 5 |
| Trichoptera | Hydrobiosidae |  | 1.80 | 1.14 | 5 |
| Trichoptera | Hydropsychidae* |  | 2.93 | 1.69 | 27 |
| Trichoptera | Lepidostomatidae |  | 1.32 | 0.48 | 15 |
| Trichoptera | Leptoceridae**†** |  | 1.55 | 1.99 | 6 |
| Trichoptera | Limnephilidae* |  | 35.55 | 40.29 | 6 |
| Trichoptera | Molannidae**†** |  | 3.01 | 0.13 | 3 |
| Trichoptera | Philopotamidae |  | 1.09 | 0.47 | 11 |
| Trichoptera | Phryganopsychidae |  | 5.50 | 2.03 | 7 |
| Trichoptera | Polycentropodidae |  | 1.29 | 0.69 | 12 |
| Trichoptera | Psychomyiidae |  | 0.59 | 0.45 | 3 |
| Trichoptera | Rhyacophilidae |  | 1.90 | 1.07 | 24 |
| Trichoptera | Stenopsychidae |  | 36.28 | 14.92 | 6 |
| Trichoptera | Uenoidae |  | 4.38 | 5.19 | 16 |
| Diptera | Chironomidae* |  | 0.17 | 0.15 | 21 |
| Diptera | Tipulidae* |  | 1.14 | 1.69 | 20 |
