# Supplementary material for: Estimates of resource transfer via winged adult insects from the hyporheic zone in a gravel‐bed river
Source: Ecol Evol. 2021 Mar 11;11(9):4656–69. doi: 10.1002/ece3.7366 (PMC8093731; doi:10.1002/ece3.7366)
Supplement: Supplementary file 5 — Appendix S5 [file ECE3-11-4656-s003.docx]

**Supplementary material S5.** Estimation of taxon-specific mean abundance for winged adults for lateral direction (A) and longitudinal direction (B)

We calculated the taxon-specific mean abundance (the number of individuals caught in Malaise trap/trapping duration, days/the number of traps used). Then, we estimated the total abundance as a sum of mean taxon-specific abundances in each temporal window. Finally, the proportion of each taxon for each temporal window was calculated from the total abundance of the same window. Chloroperlidae excluded *Alloperla ishikariana*.

Table S5(A) Calculation for lateral direction based on single-headed Malaise trap from May to August in 2019

| Order | Family | May | | | | June | | | | July | | | | August | | | |
| --- | --- | --- | --- | --- | --- | --- | --- | --- | --- | --- | --- | --- | --- | --- | --- | --- | --- |
|  |  | First | | Second | | First | | Second | | First | | Second | | First | | Second | |
|  |  | mean | proportion | mean | proportion | mean | proportion | mean | proportion | mean | proportion | mean | proportion | mean | proportion | mean | proportion |
| Ephemeroptera | Baetidae | 0.00 | 0.00 | 0.00 | 0.00 | 0.02 | 0.00 | 0.00 | 0.00 | 0.00 | 0.00 | 0.00 | 0.00 | 0.00 | 0.00 | 0.00 | 0.00 |
| Ephemeroptera | Heptageniidae | 0.00 | 0.00 | 0.00 | 0.00 | 0.02 | 0.00 | 0.00 | 0.00 | 0.00 | 0.00 | 0.00 | 0.00 | 0.00 | 0.00 | 0.00 | 0.00 |
| Plecoptera | Capniidae | 0.65 | 0.04 | 0.20 | 0.03 | 0.05 | 0.01 | 0.00 | 0.00 | 0.00 | 0.00 | 0.00 | 0.00 | 0.00 | 0.00 | 0.00 | 0.00 |
| Plecoptera | Chloroperlidae | 0.00 | 0.00 | 0.05 | 0.01 | 0.05 | 0.01 | 0.00 | 0.00 | 0.00 | 0.00 | 0.00 | 0.00 | 0.00 | 0.00 | 0.00 | 0.00 |
| Plecoptera | Leuctridae | 15.15 | 0.82 | 4.95 | 0.63 | 1.93 | 0.37 | 0.00 | 0.00 | 0.00 | 0.00 | 0.00 | 0.00 | 0.00 | 0.00 | 0.00 | 0.00 |
| Plecoptera | Neumoridae | 0.50 | 0.03 | 0.60 | 0.08 | 0.57 | 0.11 | 0.08 | 0.02 | 0.03 | 0.02 | 0.02 | 0.06 | 0.04 | 0.04 | 0.03 | 0.05 |
| Plecoptera | Perlodidae | 0.45 | 0.02 | 0.90 | 0.12 | 1.26 | 0.24 | 0.42 | 0.10 | 0.14 | 0.10 | 0.02 | 0.06 | 0.00 | 0.00 | 0.02 | 0.03 |
| Trichoptera | Apataniidae | 0.55 | 0.03 | 0.20 | 0.03 | 0.05 | 0.01 | 0.00 | 0.00 | 0.00 | 0.00 | 0.00 | 0.00 | 0.00 | 0.00 | 0.00 | 0.00 |
| Trichoptera | Glossosomatidae | 1.05 | 0.06 | 0.70 | 0.09 | 0.14 | 0.03 | 0.04 | 0.01 | 0.00 | 0.00 | 0.00 | 0.00 | 0.00 | 0.00 | 0.05 | 0.08 |
| Trichoptera | Goeridae | 0.00 | 0.00 | 0.00 | 0.00 | 0.04 | 0.01 | 0.00 | 0.00 | 0.03 | 0.02 | 0.02 | 0.06 | 0.00 | 0.00 | 0.00 | 0.00 |
| Trichoptera | Hydrobiosidae | 0.05 | 0.00 | 0.00 | 0.00 | 0.25 | 0.05 | 0.17 | 0.04 | 0.00 | 0.00 | 0.00 | 0.00 | 0.09 | 0.09 | 0.19 | 0.31 |
| Trichoptera | Hydropsychidae | 0.00 | 0.00 | 0.00 | 0.00 | 0.05 | 0.01 | 0.04 | 0.01 | 0.03 | 0.02 | 0.06 | 0.17 | 0.12 | 0.12 | 0.07 | 0.11 |
| Trichoptera | Lepidostomatidae | 0.05 | 0.00 | 0.05 | 0.01 | 0.25 | 0.05 | 0.00 | 0.00 | 0.03 | 0.02 | 0.10 | 0.28 | 0.21 | 0.21 | 0.06 | 0.10 |
| Trichoptera | Limnephilidae | 0.00 | 0.00 | 0.10 | 0.01 | 0.01 | 0.00 | 0.00 | 0.00 | 0.00 | 0.00 | 0.00 | 0.00 | 0.00 | 0.00 | 0.00 | 0.00 |
| Trichoptera | Philopotamidae | 0.00 | 0.00 | 0.00 | 0.00 | 0.32 | 0.06 | 3.46 | 0.81 | 1.06 | 0.77 | 0.14 | 0.39 | 0.51 | 0.52 | 0.17 | 0.28 |
| Trichoptera | Phryganopsychidae | 0.00 | 0.00 | 0.00 | 0.00 | 0.00 | 0.00 | 0.00 | 0.00 | 0.00 | 0.00 | 0.00 | 0.00 | 0.00 | 0.00 | 0.00 | 0.00 |
| Trichoptera | Rhyacophilidae | 0.00 | 0.00 | 0.00 | 0.00 | 0.05 | 0.01 | 0.04 | 0.01 | 0.06 | 0.04 | 0.00 | 0.00 | 0.00 | 0.00 | 0.00 | 0.00 |
| Trichoptera | Stenopsychidae | 0.00 | 0.00 | 0.05 | 0.01 | 0.10 | 0.02 | 0.00 | 0.00 | 0.00 | 0.00 | 0.00 | 0.00 | 0.02 | 0.02 | 0.01 | 0.02 |
| Trichoptera | Uenoidae | 0.00 | 0.00 | 0.00 | 0.00 | 0.00 | 0.00 | 0.00 | 0.00 | 0.00 | 0.00 | 0.00 | 0.00 | 0.00 | 0.00 | 0.01 | 0.02 |
| Total abundance | | 18.45 |  | 7.80 |  | 5.16 |  | 4.25 |  | 1.38 |  | 0.36 |  | 0.99 |  | 0.61 |  |

Table S5(B) Calculation for longitudinal direction based on hanging Malaise trap from June to October in 2017-18

| Order | Family | June | | July | | August | | | | September | | | | October | |
| --- | --- | --- | --- | --- | --- | --- | --- | --- | --- | --- | --- | --- | --- | --- | --- |
|  |  | Second | | Second | | First | | Second | | First | | Second | | Second | |
|  |  | mean | proportion | mean | proportion | mean | proportion | mean | proportion | mean | proportion | mean | proportion | mean | proportion |
| Ephemeroptera | Baetidae | 0.01 | 0.00 | 0.09 | 0.04 | 0.00 | 0.00 | 0.00 | 0.00 | 0.00 | 0.00 | 0.01 | 0.00 | 0.03 | 0.02 |
| Ephemeroptera | Caenidae | 0.00 | 0.00 | 0.00 | 0.00 | 0.00 | 0.00 | 0.00 | 0.00 | 0.13 | 0.03 | 0.00 | 0.00 | 0.00 | 0.00 |
| Ephemeroptera | Heptageniidae | 0.01 | 0.00 | 0.00 | 0.00 | 0.00 | 0.00 | 0.00 | 0.00 | 0.06 | 0.02 | 0.00 | 0.00 | 0.00 | 0.00 |
| Ephemeroptera | Leptophlebiidae | 0.01 | 0.00 | 0.00 | 0.00 | 0.00 | 0.00 | 0.00 | 0.00 | 0.00 | 0.00 | 0.02 | 0.00 | 0.00 | 0.00 |
| Plecoptera | Neumoridae | 0.38 | 0.07 | 0.05 | 0.02 | 0.01 | 0.00 | 0.00 | 0.00 | 0.69 | 0.17 | 1.00 | 0.24 | 0.42 | 0.33 |
| Plecoptera | Perlodidae | 0.03 | 0.01 | 0.06 | 0.02 | 0.01 | 0.00 | 0.00 | 0.00 | 0.00 | 0.00 | 0.00 | 0.00 | 0.00 | 0.00 |
| Trichoptera | Apataniidae | 0.16 | 0.03 | 0.00 | 0.00 | 0.01 | 0.00 | 0.00 | 0.00 | 0.06 | 0.02 | 1.09 | 0.27 | 0.55 | 0.43 |
| Trichoptera | Glossosomatidae | 0.26 | 0.05 | 0.05 | 0.02 | 0.04 | 0.01 | 0.02 | 0.03 | 0.31 | 0.08 | 0.19 | 0.05 | 0.13 | 0.10 |
| Trichoptera | Goeridae | 0.02 | 0.00 | 0.08 | 0.03 | 0.02 | 0.01 | 0.21 | 0.29 | 0.88 | 0.22 | 0.06 | 0.02 | 0.00 | 0.00 |
| Trichoptera | Hydrobiosidae | 0.14 | 0.03 | 0.03 | 0.01 | 0.03 | 0.01 | 0.02 | 0.03 | 0.00 | 0.00 | 0.12 | 0.03 | 0.04 | 0.03 |
| Trichoptera | Hydropsychidae | 0.04 | 0.01 | 0.05 | 0.02 | 0.31 | 0.09 | 0.00 | 0.00 | 0.19 | 0.05 | 0.04 | 0.01 | 0.00 | 0.00 |
| Trichoptera | Lepidostomatidae | 0.24 | 0.05 | 0.25 | 0.10 | 0.41 | 0.12 | 0.04 | 0.06 | 0.56 | 0.14 | 0.21 | 0.05 | 0.03 | 0.02 |
| Trichoptera | Leptoceridae | 0.00 | 0.00 | 0.06 | 0.02 | 0.09 | 0.03 | 0.00 | 0.00 | 0.19 | 0.05 | 0.20 | 0.05 | 0.03 | 0.02 |
| Trichoptera | Limnephilidae | 0.00 | 0.00 | 0.02 | 0.01 | 0.01 | 0.00 | 0.13 | 0.18 | 0.00 | 0.00 | 0.55 | 0.14 | 0.04 | 0.03 |
| Trichoptera | Molannidae | 0.00 | 0.00 | 0.00 | 0.00 | 0.00 | 0.00 | 0.00 | 0.00 | 0.00 | 0.00 | 0.04 | 0.01 | 0.00 | 0.00 |
| Trichoptera | Philopotamidae | 3.67 | 0.70 | 1.86 | 0.71 | 2.19 | 0.64 | 0.25 | 0.35 | 0.19 | 0.05 | 0.07 | 0.02 | 0.00 | 0.00 |
| Trichoptera | Phryganopsychidae | 0.02 | 0.00 | 0.00 | 0.00 | 0.00 | 0.00 | 0.00 | 0.00 | 0.31 | 0.08 | 0.34 | 0.08 | 0.01 | 0.01 |
| Trichoptera | Polycentropodidae | 0.00 | 0.00 | 0.00 | 0.00 | 0.09 | 0.03 | 0.00 | 0.00 | 0.00 | 0.00 | 0.01 | 0.00 | 0.00 | 0.00 |
| Trichoptera | Rhyacophilidae | 0.09 | 0.02 | 0.00 | 0.00 | 0.01 | 0.00 | 0.00 | 0.00 | 0.25 | 0.06 | 0.03 | 0.01 | 0.00 | 0.00 |
| Trichoptera | Stenopsychidae | 0.14 | 0.03 | 0.02 | 0.01 | 0.17 | 0.05 | 0.04 | 0.06 | 0.19 | 0.05 | 0.13 | 0.03 | 0.00 | 0.00 |
| Total abundance | | 5.21 |  | 2.61 |  | 3.41 |  | 0.71 |  | 4.00 |  | 4.10 |  | 1.28 |  |
