# Supplementary material for: Estimates of resource transfer via winged adult insects from the hyporheic zone in a gravel‐bed river
Source: Ecol Evol. 2021 Mar 11;11(9):4656–69. doi: 10.1002/ece3.7366 (PMC8093731; doi:10.1002/ece3.7366)
Supplement: Supplementary file 6 — Appendix S6 [file ECE3-11-4656-s005.docx]

**Supplementary material S6.** Estimation of taxon-specific mean dry biomass for winged adults for lateral direction (A) and longitudinal direction (B)

Taxon-specific individual dry biomass (mg) estimated partially based on weighted mean biomass using information provided in Supplementary materials S4 and S5. The values were estimated for first and second halves for the available period between May and October in the Satsunai River, Hokkaido, Japan. Plecoptera without species names denote non-hyporheic Plecoptera that excludes *Alloperla ishikariana*. Mean dry biomass was used for *A. ishikariana* for all windows.

Table S6(A) Calculation for lateral direction based on single-headed Malaise trap records. *Because no individuals were collected for these windows in 2019, zero values were provided; these were replaced with mean individual biomass for Ephemeroptera provided in Supplementary material S4 (2.72 mg) when estimating biomass of Ephemeroptera for these windows. **Due to lack of data in first half of May, data in second half of May was used instead

| Order | Species | May | | June | | July | | August | |
| --- | --- | --- | --- | --- | --- | --- | --- | --- | --- |
|  |  | First** | Second | First | Second | First | Second | First | Second |
| Ephemeroptera |  | 0* | 0* | 0.03 | 0* | 0* | 0* | 0* | 0* |
| Plecoptera |  | 4.37 | 4.37 | 4.75 | 0.95 | 1.03 | 0.91 | 0.06 | 0.47 |
| Plecoptera | *A. ishikariana* | 2.07 | 2.07 | 2.07 | 2.07 | 2.07 | 2.07 | 2.07 | 2.07 |
| Trichoptera |  | 2.68 | 2.68 | 2.02 | 1.05 | 1.11 | 1.35 | 2.05 | 2.18 |

Table S6(B) Calculation for longitudinal direction based on hanging Malaise trap records. *Because no individuals were collected for these windows in 2017-18, zero values were provided; these were replaced with mean individual biomass for Ephemeroptera and Plecoptera provided in Supplementary material S4 (2.72 and 3.48 mg, respectively) when estimating biomass of Ephemeroptera and Plecoptera for these windows. **Due to lack of data in first half of June, July and October, data in second half was used instead for these months

| Order | Species | June | | July | | August | | September | | October | |
| --- | --- | --- | --- | --- | --- | --- | --- | --- | --- | --- | --- |
|  |  | First** | Second | First** | Second | First | Second | First | Second | First** | Second |
| Ephemeroptera |  | 0.008 | 0.008 | 0.05 | 0.05 | 0* | 0* | 0.06 | 0.01 | 0.03 | 0.03 |
| Plecoptera |  | 0.17 | 0.17 | 0.44 | 0.44 | 0.07 | 0* | 0.20 | 0.28 | 0.37 | 0.37 |
| Plecoptera | *A. ishikariana* | 2.07 | 2.07 | 2.07 | 2.07 | 2.07 | 2.07 | 2.07 | 2.07 | 2.07 | 2.07 |
| Trichoptera |  | 2.11 | 2.11 | 1.60 | 1.60 | 3.20 | 10.22 | 3.78 | 7.43 | 2.25 | 2.25 |
