# Supplementary material for: Estimates of resource transfer via winged adult insects from the hyporheic zone in a gravel‐bed river
Source: Ecol Evol. 2021 Mar 11;11(9):4656–69. doi: 10.1002/ece3.7366 (PMC8093731; doi:10.1002/ece3.7366)
Supplement: Supplementary file 7 — Appendix S7 [file ECE3-11-4656-s008.docx]

**Supplementary material S7.** Estimation of mean returning rates of Ephemeroptera (E), Plecoptera (P), Trichoptera (T), and Diptera (D) to the channel based on past studies

For the estimation of how much biomass moved back to the channel, we used taxa informaiton from previous reports of Jackson and Fisher (1986); Gray (1989); Stagliano, Benke and Anderson (1998) and Francis, Schindler and Moore (2006). These estimation overall included 13 out of 15 families found in the Satsunai River, Hokkaido, Japan (see S4). We acquired the returning rate (%) data for each taxa from the studies [except for Gray (1989) where we were able to get one mean returning rate for all orders] and pooled together the order-level data and finally calculated the mean returning rates for E, P, T, and D.

| References | Studied taxa | Period of study | Order-wise returning rate (%) | | | |
| --- | --- | --- | --- | --- | --- | --- |
|  |  |  | E | P | T | D |
| Jackson and Fisher (1986) | *Baetis quilleri* and *Baetis insignificans* (E), *Tricorythodes dimorphus* (P), *Helicopsyche mexicana* (T) | 1 June 1982 to 30 September 1983 | 1.3 | 20.5 | 2.5 | - |
|  | *Leptohyphes packeri* (E)*, Cheumatopsyche arizonensis* (T) |  | 3.1 | - | 0 | - |
|  | Chironomidae (D) |  | - | - | - | 1.4 |
|  | *Cryptolabis* sp. (D) |  | - | - | - | 12.5 |
| Gray (1989) | *Fallceon quilleri* (E), *Caenis delicata* (E), *Stenonema femoratum* (E), *Stenacron interpunctatum*(E), *Choroterpes* sp.*(E), Perlesta placida* (P), *Zealeuctra* sp. (P), *Hydroptila* sp. (T), *Cheumatopsyche pettiti* (T), Chironomidae (D), Tipulidae (D), Simuliidae (D), and Ceratopogonidae (D) | 31 May 1986 to 8 August 1987 (various sampling days for 24h period) | 0.4 | 0.4 | 0.4 | 0.4 |
| Stagliano, Benke and Anderson (1998) | Hydroptilidae (T) | December 1992 to December 1993 | - | - | 19.2 | - |
| Francis, Schindler and Moore (2006) | *Baetis* (E), *Drunella* (E), *Cinygmula* (E), Chloroperlidae (P), Nemouridae (P), Perlodidae (P), *Ecclisomyia* (T) | 26 June to 24 July in 2004 | 4.7 | 22.9 | 10.9 | - |
|  |  | Mean | 2.4 | 14.6 | 6.6 | 4.8 |

References

Francis, T. B., Schindler, D. E. and Moore, J. W. (2006) ‘Aquatic insects play a minor role in dispersing salmon-derived nutrients into riparian forests in southwestern Alaska’, *Canadian Journal of Fisheries and Aquatic Sciences*, 63(11), pp. 2543–2552. doi: 10.1139/F06-144.

Gray, L. J. (1989) ‘Emergence production and export of aquatic insects from a tallgrass prairie stream’, *The Southwestern Naturalist*, 34(3), pp. 313–318. doi: 10.2307/3672158.

Jackson, J. K. and Fisher, S. G. (1986) ‘Secondary production, emergence, and export of aquatic insects of a Sonoran desert stream’, *Ecology*, 67(3), pp. 629–638.

Stagliano, D. M., Benke, A. C. and Anderson, D. H. (1998) ‘Emergence of aquatic insects from 2 habitats in a small wetland of the southeastern USA: Temporal patterns of numbers and biomass’, *Journal of the North American Benthological Society*, 17(1), pp. 37–53. doi: 10.2307/1468050.
