# Supplementary material for: Estimates of resource transfer via winged adult insects from the hyporheic zone in a gravel‐bed river
Source: Ecol Evol. 2021 Mar 11;11(9):4656–69. doi: 10.1002/ece3.7366 (PMC8093731; doi:10.1002/ece3.7366)
Supplement: Supplementary file 8 — Appendix S8 [file ECE3-11-4656-s004.docx]

**Supplementary material S8.** Calculation of taxon-specific trap conversion factors

For Ephemeroptera (E), non-hyporheic Plecoptera (P) (Plecoptera without *Alloperla ishikariana*), Trichoptera (T), and *A. ishikariana*, we first calculated the relative abundances for unit trapping area (m^2^) for each sampling event for single-headed Malaise (SM) trap and hanging Malaise (HM) trap and obtained the mean relative abundances for each trap by averaging for each sampling event; finally, we divided the mean relative abundances of SM trap by HM trap to calculate the trap conversion factor *a* and HM trap by SM trap to calculate the trap conversion factor *b*. SM and HM traps were placed at the river edge in the riparian forest in parallel with the river in L2 site on July 2018, and in L2, L7, L8 and L9_1 sites on June 2019. NA - not applicable.

| Starting date | Collection date | Site | Ephemeroptera | | | | | | | Plecoptera without *A. ishikariana* | | | | | | |
| --- | --- | --- | --- | --- | --- | --- | --- | --- | --- | --- | --- | --- | --- | --- | --- | --- |
|  |  |  | Trap abundance | | Abundance per m^2^ | | Total | Relative abundance | | Trap abundance | | Abundance per m^2^ | | Total | Relative abundance | |
|  |  |  | SM | HM | SM | HM |  | SM | HM | SM | HM | SM | HM |  | SM | HM |
| 4-Jul-18 | 6-Jul-18 | L2 | 0 | 0 | 0 | 0 | 0 | NA | NA | 0 | 3 | 0 | 0.75 | 0.75 | 0 | 1 |
| 4-Jul-18 | 6-Jul-18 | L2 | 0 | 0 | 0 | 0 | 0 | NA | NA | 1 | 0 | 0.37 | 0 | 0.37 | 1 | 0 |
| 6-Jul-18 | 10-Jul-18 | L2 | 0 | 2 | 0 | 0.50 | 0.50 | 0 | 1 | 5 | 20 | 1.84 | 4.97 | 6.81 | 0.27 | 0.73 |
| 6-Jul-18 | 10-Jul-18 | L2 | 0 | 1 | 0 | 0.25 | 0.25 | 0 | 1 | 1 | 31 | 0.37 | 7.70 | 8.07 | 0.05 | 0.95 |
| 10-Jul-18 | 23-Jul-18 | L2 | 0 | 0 | 0 | 0 | 0 | NA | NA | 6 | 10 | 2.21 | 2.48 | 4.69 | 0.47 | 0.53 |
| 10-Jul-18 | 23-Jul-18 | L2 | 0 | 0 | 0 | 0 | 0 | NA | NA | 1 | 4 | 0.37 | 0.99 | 1.36 | 0.27 | 0.73 |
| 10-Jun-19 | 13-Jun-19 | L9_1 | 0 | 0 | 0 | 0 | 0 | NA | NA | 19 | 16 | 6.99 | 3.98 | 10.96 | 0.64 | 0.36 |
| 13-Jun-19 | 20-Jun-19 | L9_1 | 0 | 1 | 0 | 0.25 | 0.25 | 0 | 1 | 117 | 12 | 43.04 | 2.98 | 46.02 | 0.94 | 0.06 |
| 20-Jun-19 | 22-Jun-19 | L9_1 | 0 | 0 | 0 | 0 | 0 | NA | NA | 0 | 1 | 0 | 0.25 | 0.25 | 0 | 1 |
| 22-Jun-19 | 24-Jun-19 | L9_1 | 0 | 0 | 0 | 0 | 0 | NA | NA | 4 | 7 | 1.47 | 1.74 | 3.21 | 0.46 | 0.54 |
| 10-Jun-19 | 13-Jun-19 | L8 | 0 | 5 | 0 | 1.24 | 1.24 | 0 | 1 | 2 | 9 | 0.74 | 2.24 | 2.97 | 0.25 | 0.75 |
| 13-Jun-19 | 20-Jun-19 | L8 | 1 | 13 | 0.37 | 3.23 | 3.60 | 0.10 | 0.90 | 5 | 29 | 1.84 | 7.20 | 9.04 | 0.20 | 0.80 |
| 20-Jun-19 | 22-Jun-19 | L8 | 0 | 1 | 0 | 0.25 | 0.25 | 0 | 1 | 1 | 2 | 0.37 | 0.50 | 0.86 | 0.43 | 0.57 |
| 22-Jun-19 | 24-Jun-19 | L8 | 0 | 3 | 0 | 0.75 | 0.75 | 0 | 1 | 1 | 4 | 0.37 | 0.99 | 1.36 | 0.27 | 0.73 |
| 10-Jun-19 | 13-Jun-19 | L7 | 0 | 0 | 0 | 0 | 0 | NA | NA | 4 | 4 | 1.47 | 0.99 | 2.47 | 0.60 | 0.40 |
| 13-Jun-19 | 20-Jun-19 | L7 | 0 | 0 | 0 | 0 | 0 | NA | NA | 4 | 10 | 1.47 | 2.48 | 3.96 | 0.37 | 0.63 |
| 20-Jun-19 | 24-Jun-19 | L7 | 0 | 0 | 0 | 0 | 0 | NA | NA | 0 | 3 | 0 | 0.75 | 0.75 | 0 | 1 |
| 10-Jun-19 | 13-Jun-19 | L2 | 0 | 3 | 0 | 0.75 | 0.75 | 0 | 1 | 0 | 8 | 0 | 1.99 | 1.99 | 0 | 1 |
| 13-Jun-19 | 20-Jun-19 | L2 | 0 | 6 | 0 | 1.49 | 1.49 | 0 | 1 | 2 | 34 | 0.74 | 8.45 | 9.18 | 0.08 | 0.92 |
| 20-Jun-19 | 24-Jun-19 | L2 | 0 | 1 | 0 | 0.25 | 0.25 | 0 | 1 | 0 | 11 | 0 | 2.73 | 2.73 | 0 | 1 |
| Mean relative proportional abundance per m^2^ | | | - | - | - | - | - | 0.01 | 0.99 | - | - | - | - | - | 0.31 | 0.69 |
| **Trap conversion factor *a*** | | | **-** | **-** | **-** | **-** | **-** | **0.01** | | **-** | **-** | **-** | **-** | **-** | **0.46** | |
| **Trap conversion factor *b*** | | | **-** | **-** | **-** | **-** | **-** | **96.80** | | **-** | **-** | **-** | **-** | **-** | **2.18** | |

*to be continued…*

| Starting date | Collection date | Site | Trichoptera | | | | | | | *A. ishikariana* | | | | | | |
| --- | --- | --- | --- | --- | --- | --- | --- | --- | --- | --- | --- | --- | --- | --- | --- | --- |
|  |  |  | Trap abundance | | Abundance per m^2^ | | Total | Relative abundance | | Trap abundance | | Abundance per m^2^ | | Total | Relative abundance | |
|  |  |  | SM | HM | SM | HM |  | SM | HM | SM | HM | SM | HM |  | SM | HM |
| 4-Jul-18 | 6-Jul-18 | L2 | 0 | 5 | 0 | 1.24 | 1.24 | 0 | 1 | 3 | 0 | 1.10 | 0 | 1.10 | 1 | 0 |
| 4-Jul-18 | 6-Jul-18 | L2 | 1 | 1 | 0.37 | 0.25 | 0.62 | 0.60 | 0.40 | 2 | 0 | 0.74 | 0 | 0.74 | 1 | 0 |
| 6-Jul-18 | 10-Jul-18 | L2 | 0 | 35 | 0 | 8.70 | 8.70 | 0 | 1 | 1 | 0 | 0.37 | 0 | 0.37 | 1 | 0 |
| 6-Jul-18 | 10-Jul-18 | L2 | 4 | 32 | 1.47 | 7.95 | 9.42 | 0.16 | 0.84 | 2 | 15 | 0.74 | 3.73 | 4.46 | 0.16 | 0.84 |
| 10-Jul-18 | 23-Jul-18 | L2 | 4 | 18 | 1.47 | 4.47 | 5.94 | 0.25 | 0.75 | 3 | 5 | 1.10 | 1.24 | 2.35 | 0.47 | 0.53 |
| 10-Jul-18 | 23-Jul-18 | L2 | 9 | 3 | 3.31 | 0.75 | 4.06 | 0.82 | 0.18 | 7 | 1 | 2.57 | 0.25 | 2.82 | 0.91 | 0.09 |
| 10-Jun-19 | 13-Jun-19 | L9_1 | 28 | 69 | 10.30 | 17.14 | 27.44 | 0.38 | 0.62 | 2 | 21 | 0.74 | 5.22 | 5.95 | 0.12 | 0.88 |
| 13-Jun-19 | 20-Jun-19 | L9_1 | 10 | 348 | 3.68 | 86.46 | 90.14 | 0.04 | 0.96 | 9 | 22 | 3.31 | 5.47 | 8.78 | 0.38 | 0.62 |
| 20-Jun-19 | 22-Jun-19 | L9_1 | 3 | 20 | 1.10 | 4.97 | 6.07 | 0.18 | 0.82 | 2 | 3 | 0.74 | 0.75 | 1.48 | 0.50 | 0.50 |
| 22-Jun-19 | 24-Jun-19 | L9_1 | 4 | 13 | 1.47 | 3.23 | 4.70 | 0.31 | 0.69 | 1 | 22 | 0.37 | 5.47 | 5.83 | 0.06 | 0.94 |
| 10-Jun-19 | 13-Jun-19 | L8 | 32 | 244 | 11.77 | 60.62 | 72.39 | 0.16 | 0.84 | 1 | 32 | 0.37 | 7.95 | 8.32 | 0.04 | 0.96 |
| 13-Jun-19 | 20-Jun-19 | L8 | 77 | 832 | 28.32 | 206.71 | 235.03 | 0.12 | 0.88 | 3 | 59 | 1.10 | 14.66 | 15.76 | 0.07 | 0.93 |
| 20-Jun-19 | 22-Jun-19 | L8 | 2 | 495 | 0.74 | 122.98 | 123.72 | 0.01 | 0.99 | 2 | 15 | 0.74 | 3.73 | 4.46 | 0.16 | 0.84 |
| 22-Jun-19 | 24-Jun-19 | L8 | 6 | 122 | 2.21 | 30.31 | 32.52 | 0.07 | 0.93 | 0 | 9 | 0 | 2.24 | 2.24 | 0 | 1 |
| 10-Jun-19 | 13-Jun-19 | L7 | 2 | 8 | 0.74 | 1.99 | 2.72 | 0.27 | 0.73 | 0 | 6 | 0 | 1.49 | 1.49 | 0 | 1 |
| 13-Jun-19 | 20-Jun-19 | L7 | 11 | 79 | 4.05 | 19.63 | 23.67 | 0.17 | 0.83 | 7 | 9 | 2.57 | 2.24 | 4.81 | 0.54 | 0.46 |
| 20-Jun-19 | 24-Jun-19 | L7 | 4 | 28 | 1.47 | 6.96 | 8.43 | 0.17 | 0.83 | 0 | 6 | 0 | 1.49 | 1.49 | 0 | 1 |
| 10-Jun-19 | 13-Jun-19 | L2 | 30 | 329 | 11.04 | 81.74 | 92.77 | 0.12 | 0.88 | 1 | 24 | 0.37 | 5.96 | 6.33 | 0.06 | 0.94 |
| 13-Jun-19 | 20-Jun-19 | L2 | 75 | 492 | 27.59 | 122.24 | 149.82 | 0.18 | 0.82 | 9 | 33 | 3.31 | 8.20 | 11.51 | 0.29 | 0.71 |
| 20-Jun-19 | 24-Jun-19 | L2 | 44 | 294 | 16.19 | 73.04 | 89.23 | 0.18 | 0.82 | 3 | 17 | 1.10 | 4.22 | 5.33 | 0.21 | 0.79 |
| Mean relative proportional abundance per m^2^ | | | - | - | - | - | - | 0.21 | 0.79 | - | - | - | - | - | 0.35 | 0.65 |
| **Trap conversion factor *a*** | | | **-** | **-** | **-** | **-** | **-** | **0.26** | |  |  |  |  |  | **0.54** | |
| **Trap conversion factor *b*** | | | **-** | **-** | **-** | **-** | **-** | **3.78** | |  |  |  |  |  | **1.87** | |
